# Thecaloscopy Reduces the Risk of Recurrent Perineural (Tarlov) Cysts after Microsurgical Resection

**DOI:** 10.3390/neurolint16020033

**Published:** 2024-04-17

**Authors:** Michael Luchtmann, Angelika Klammer, Mircea-Alin Iova, André Roth, Vijay Kumar Chanamolu, Christian Mawrin, Jan-Peter Warnke

**Affiliations:** 1 Department of Neurosurgergy, Heinrich-Braun-Klinikum, 08060 Zwickau, Germany; 2 Department of Neurosurgery, Paracelsus-Klinikum, 08060 Zwickau, Germany; 3 Leupold Institute for Applied Natural Sciences, University of Applied Science, 08056 Zwickau, Germany; 4 Vigdis Thompson Foundation, 08412 Werdau, Germany; 5 Department of Neuropathology, Otto-von-Guericke University, 39120 Magdeburg, Germany

**Keywords:** thecaloscopy, leptomeningeopathy, Tarlov cyst, perineurial cyst

## Abstract

Sacral Tarlov cysts (TCs), often asymptomatic, can cause significant pain and severe neurological dysfunction. Conventional treatments are generally associated with high recurrence and complication rates. Specifically, the substantial recurrence rates, which can reach as high as 50%, significantly impact long-term outcomes. Recent evidence increasingly supports the hypothesis that the formation of Tarlov cysts (TCs) may be associated with inflammatory processes within the nerve root sheath, further exacerbated by elevated cerebrospinal fluid (CSF) pressure. This retrospective study explores thecaloscopy, combined with surgical techniques, as a more effective alternative. We observed a total of 78 patients, 48 of whom underwent endoscopic fenestration of the arachnoid sheath in addition to microsurgical resection of the TC. We found that the fenestration of the arachnoid sheath at the level of lumbosacral spinal nerve root entry led to a significantly decreased risk of developing recurrent TCs (5/48 vs. 9/30). Only one of the patients suffered from a persistent new bladder dysfunction after microsurgical resection. This presented technique provides a promising treatment path for the future management of TCs, offering a safe and more effective treatment option compared to previous methods. Additionally, the advantages of the thecaloscopy provide pathophysiological implications regarding the development of perineural cysts.

## 1. Introduction

Perineural cysts, named after their first describer Isadore M. Tarlov, also known as Tarlov cysts (TCs), predominantly emerging from augmented cerebrospinal fluid (CSF) aggregations situated between the endoneurium and perineurium of sacral spinal nerve roots, demonstrate a significant occurrence rate of approximately 5% in the adult cohort [1,2,3,4]. Despite this prevalence, most TCs are asymptomatic and are often serendipitously detected through radiological evaluations of the lumbosacral spine. The medical consensus is that these asymptomatic cysts do not necessitate any kind of treatment or intervention. Nonetheless, TCs can, in certain scenarios, induce severe clinical symptoms such as persistent radicular pain and dysfunctions pertaining to bladder, bowel, and sexual health [5]. Despite mounting evidence, the clinical relevance of TCs remains a contentious subject, with some sectors of the medical community regarding them as negligible even in the face of characteristic symptomatic presentations [6,7]. The treatment landscape for TCs is diverse, encompassing a spectrum from microsurgical techniques to percutaneous strategies and adhesive applications. Surgical methodologies vary, including procedures like cyst diminution, fenestration, excision, imbrication, clipping, and cyst shunting, executed with or without the incorporation of sacral laminoplasty [8,9,10,11]. Notably, these interventions are associated with complication rates up to 39% and recurrence probabilities as high as 50% [10,12].

This high rate of complications and recurrences has led various researchers to hypothesize that TC formation may be linked to inflammatory activities within the nerve root sheath, compounded by heightened CSF pressure [13,14]. This wide array of therapeutic options, as discussed by Naderi [15], seemingly reflects a prevailing uncertainty regarding both the most effective treatment regimen and the detailed pathophysiology of TCs [16,17].

In a recent study, we delineated both clinical and pathophysiological findings from an observational research focused on the surgical management of symptomatic TCs [18]. The procedure for patients encompassed microsurgical treatment of TCs, combined with thecaloscopy to identify potential inflammatory markers at the lumbar leptomeningeal sheath. Thecaloscopy is a minimally invasive diagnostic and therapeutic procedure that involves the direct visualization of the thecal sac and its contents using a flexible endoscope. This technique allows for the examination of the spinal canal, nerve roots, and the dural sac to diagnose and sometimes treat conditions affecting these areas. Regarding TCs, an internal shunt was created from the subarachnoid to the subdural space through fenestration of the arachnoid sheath to allow the reconstruction of the lumbar CSF flow regulation capacity by re-opening of the arachnoid nerve root perforations. In this study, we investigated the extent to which fenestration of the subarachnoid space by thecaloscopy led to a reduction in the risk of developing recurrent Tarlov cysts.

## 2. Patients and Methods

The ethics committee of the Medical Faculty of the University of Magdeburg approved the study in compliance with national legislation and the Code of Ethical Principles for Medical Research Involving Human Subjects of the World Medical Association (Declaration of Helsinki). Written informed consent was obtained from all subjects prior to the data collection and analysis.

**Figure 1:**
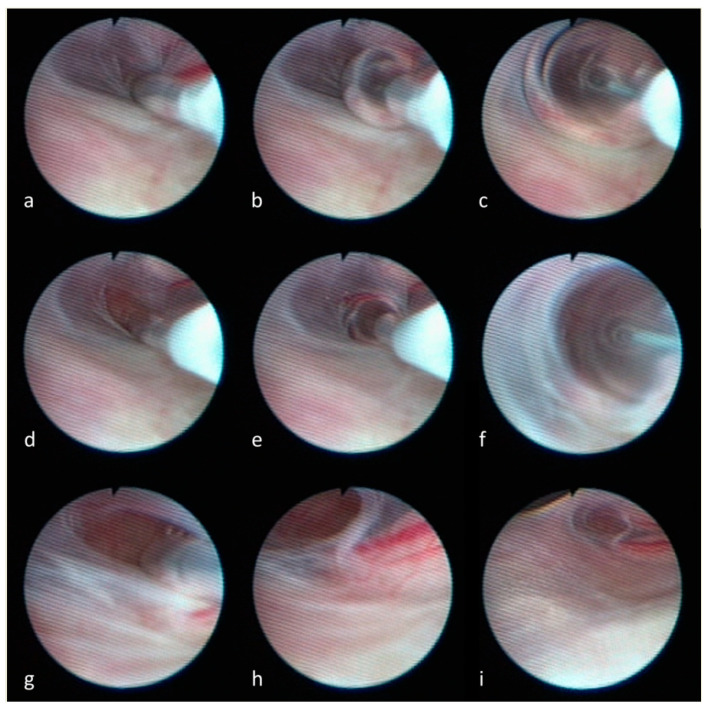
Depicted is the fenestration of the arachnoid sheath. Utilizing a flexible endoscope, in the first step the arachnoid layer is perforated with the tip of the balloon catheter (**a**). The balloon is inflated (**b**,**c**) to obtain a small opening of the arachnoid sheath (**d**). After advancing the catheter into the opening (**e**), the repeated inflation of the balloon catheter (**f**) leads to the final fenestration of the arachnoid layer (**g**,**h**) between the ventral and dorsal root of the spinal nerve (**i**).

In this retrospective study, the medical data of 78 patients who underwent surgery for symptomatic TCs between 2018 and 2021 were analyzed. All patients were recruited through the Vigdis Thompson Foundation (https://www.vigdis-thompson-foundation.org/en/), a non-profit organization dedicated to research into Tarlov cysts and rare leptomeningopathies. In our clinical routine, TCs were removed applying microsurgical techniques. As demonstrated in Figure 1, in 48 of the 78 patients, an additional thecaloscopy was performed to re-open the arachnoid nerve root perforations and to fenestrate the ventral arachnoid layers [18]. In the other 30 patients, a thecaloscopy was not feasible due to anatomical conditions (e.g., tightened space due to spinal stenosis) or previous surgical procedures (e.g., lumbar spondylodesis).

Within three days after surgery, in all patients, magnetic resonance imaging (MRI) of the lumbosacral spine was conducted to obtain a baseline for follow-up imaging. MRI examinations and the recording of clinical parameters were carried out as part of the annual follow-up examinations. Demographic data (age, gender, height, weight), clinical parameters (e.g., duration of symptoms, pain level, postoperative relief of symptoms), and characteristics of the resected TCs (e.g., size, number, bone erosion) were gathered (see Table 2). The main question was whether cysts developed again at the previously operated sites in the postoperative course. For this purpose, the MRI images were evaluated independently by two senior neurosurgeons.

Statistical analyses were performed using the software SAS 9.4 University Edition (SAS Institute, Inc., Cary, NY, USA), and since the present analysis was performed in an explorative sense, deliberately reviewed to the full level of significance. Each *p*-value < 0.05 thus represents a statistically significant result. For unadjusted analyses, Fisher’s exact tests were applied for categorical variables, and the robust *t*-test (Satterthwaite) was applied for continuous variables.

## 3. Results

The observed cohort comprised a total of 78 patients. Of these, 80.8% were female. All patients suffered from unbearable pain that could not be treated conservatively. In 48 out of 78 patients, a thecaloscopy was feasible and therefore performed prior to resection of the TCs. The remaining 30 patients were treated by microsurgical resection only [18]. The mean follow-up of all patients was 19.01 ± 12.17 months. As seen in Table 1, only 10.4% of the patients treated with an additional fenestration of the arachnoid sheath developed recurrent perineural cysts. In contrast, patients in whom a thecaloscopy was not possible suffered from significantly more recurrent TCs (5/48 vs. 9/30, *p* = 0.0368).

Table 2 reveals that most of the observed demographic, clinical as well as cyst parameters were not associated with the development of recurrent cysts. However, patients with recurrent cysts were significantly taller than patients without (175.0 ± 7.2 cm vs. 169.2 ± 7.1 cm, *p* = 0.007). Although all patients benefit from the surgical procedure, it was evident that patients with recurrent TCs suffered from more pain; overall, symptoms did not improve in the follow-up to the same extent as in patients without recurrent perineural cysts. An additional factor that has been found to be associated with re-occurrence after microsurgical resection was the bilateral growth of TCs. More than 90% of the TCs that reoccurred were bilateral. However, in more than 50% of the patients who did not develop recurrent TCs, bilateral growths were found also.

As seen in Table 2, more than 40% of the patients (32/78) suffered from bladder disturbances prior to the surgical procedure. Of the remaining patients, eight suffered from temporary worsening of bladder symptoms, which resolved within the hospital stay in seven cases. Only one of the patients suffered from persistent new bladder dysfunction after microsurgical resection. Postoperative MRI could not reveal a specific cause that would have led to an operative revision. Additionally, evidence of an asymptomatic cerebrospinal fluid fistula was found in one patient during the routine postoperative MRI. This condition did not necessitate any intervention and was no longer evident in the MRI follow-up conducted one year later.

## 4. Discussion

This retrospective approach tried to investigate whether creating an internal shunt from the spinal subarachnoid to the subdural space through fenestration of the AS applying an additional thecaloscopy may lead to a reduced risk of developing recurrent Tarlov cysts after microsurgical resection.

As shown in Table 2, thecaloscopy led to a significantly reduced occurrence of recurrent TCs. Previous work showed recurrence rates of over 50% [10]. A recurrence rate of about 10%, as observed in our thecaloscopy group, is at the lower end of the published range. Of the patients who were not treated with an additional thecaloscopy, 30% developed recurrent TCs. These results are in line with reported recurrence rates of more than 50% [19]. However, the comparison to previous reported results is quite difficult, since the indication criteria for surgical treatment as well as the surgical techniques vary tremendously across the reported studies and case reports. To our knowledge, we reported the largest observation so far with 78 patients. For the first time, we analyzed the advantage of thecaloscopy showing that the fenestration of the arachnoid sheath in addition to the microsurgical resection of the TC leads to a decreased risk of developing TCs.

In our cohort, 80.8% of all patients suffering from TCs were female. The disproportionate occurrence is already known and may be attributed to gender-specific differences in the structure of the dura mater and spinal nerve roots [7]. A study examining the perfusion and permeability of nerve roots and dorsal ganglia in patients with neuropathic pain revealed that women exhibited significantly higher permeability and interstitial leakage within the dorsal root ganglia compared to men. This increased permeability in the female anatomy potentially makes the sensory fibers and neurons within the dorsal ganglion more prone to structural affections from mechanical pressure or harmful substances, potentially leading to chronic neuropathic pain. This could be a contributing factor to the higher incidence of symptomatic TCs in women [20].

While there are many theories, the exact cause of TCs is still not clear. A widely accepted theory suggests that TCs form due to the combined effect of the hydrostatic and pulsatile forces of cerebrospinal fluid (CSF), along with a ball valve mechanism that continuously expands the cyst. This theory is supported by several findings. Thus, TCs typically appear symmetrically along nerve roots rather than following a top-down or gravity-related pattern of distribution, as indicated in recent works [7,14]. Additionally, these cysts are not easily compressed during surgery [21,22], remain inflated even after draining CSF from the neighboring thecal sac [23], and show delayed contrast uptake in myelograms [2], suggesting TCs have an less limited inflow but restricted outflow of CSF. Furthermore, the often observed sacral scalloping, pedicle erosion, and enlargement of the foramina in TC cases suggest that the CSF pressure inside these cysts is higher than normal pressures in the thecal sac [24]. Additionally, it has been hypothesized that spinal meningeal cysts arise from an inflexibility in the subarachnoid space, which fails to adequately adjust to changes in CSF volume, indicative of abnormal CSF dynamics [25]. There have been several documented instances where increased intracranial pressure is associated with TCs, although not all such patients meet the criteria for idiopathic intracranial hypertension [7,26]. It is well established that reducing hydrostatic pressure through procedures such as external lumbar CSF drainage or acetazolamide administration can alleviate symptoms of TCs and, in some cases, even diminish their size [13,26,27]. The positional shift from lying supine to standing or sitting upright causes a downward movement of CSF, leading to an increased CSF volume in the spinal dural sac and creating a caudo-rostral pressure gradient [28]. These hypotheses are in line with our findings that patients suffering from recurrent TCs were significantly larger than patients without TCs, indicating a hydrostatic component in the pathophysiology of the development of TCs. Nevertheless, the precise cause of increased hydrostatic pressure remains unclear, but genetic factors seem to be involved. As already mentioned above, a significant portion of patients with sacral TCs are women, and familial clusters have been reported [29]. Additionally, TCs are more commonly found in individuals with genetic soft tissue disorders such as hypermobility-type Ehlers–Danlos syndrome (EDS) and Marfan syndrome [30,31]. The propensity for nerve root sheath dilation in these patients, likely due to soft tissue weakness, and the frequent occurrence of increased intracranial pressure in EDS patients, support this association. To our knowledge, none of our patients suffered from EDS, Marfan syndrome or other soft tissue disturbances.

As already mentioned, the factors leading to the development of TCs are not well-known. Even the precise mechanisms of CSF circulation and its disturbances, particularly the absorption, remains poorly elucidated. However, it is well-established that CSF is absorbed via spinal nerve sheaths. Absorption sides were identified on meningeal sheaths, particularly the meningeal recesses of spinal nerve roots [32]. The proximity of TCs to the dorsal root ganglion may be related to the increased permeability of the blood–nerve interface at the dorsal root ganglion compared to other nerve roots [33]. Consequently, when hydrostatic pressure within the nerve root sheath rises, CSF may start to seep between the endoneurium and perineurium [20]. As we have shown in a recent work, we found histological signs of leptomeningeal inflammation in form of an aseptic arachnoiditis [18]. We hypothesize that the combination of inflammatory processes which lead to inflexibility in the subarachnoid space and an increased hydrostatic pressure of CSF may result in the above-mentioned ball valve mechanism. Our rationale was to dissolve the valve mechanism using balloon catheter technique as described [18] to allow the reconstruction of the lumbosacral CSF flow regulation capacity by re-opening of the arachnoid nerve root perforations and to dilatate the space between the endo- and epineurium at the level of the entry zone of the spinal nerve root.

As detailed in the results section, only one patient experienced a new postoperative bladder dysfunction, for which, despite thorough diagnostics, we were unable to determine a specific cause like a postoperative hemorrhage. The only cerebrospinal fluid fistula observed was asymptomatic and resolved spontaneously. As reported in the literature, the most prevalent complication of surgical treatment for TCs is sciatica [10]. However, in our cohort, none of the patients reported postoperative sciatica. In fact, in all cases where preoperative leg radiating pain was present (20/78), it improved following the surgery. Overall, we therefore regard the presented combined approach as a relatively safe surgical procedure.

However, some limiting aspects beyond the obvious flaws of retrospective study designs, such as retrospective data set, possible incomplete data, etc., must be discussed. Since most TCs are asymptomatic it is not clear how recurrent perineural cysts contribute to the remaining symptoms of the patients. The same applies to the factors described, which led to us not applying thecaloscopy. Additionally, due to the clinical routine follow-up MR imaging was performed annually. We had no information on how quickly TCs reoccurred. To address these limitations (particularly the lack of objective measures of the complaints and symptoms), a prospective study is already enrolled.

## Figures and Tables

**Table 1:** Utilization of thecaloscopy led to a significant reduction in recurrent cysts after microsurgical treatment.

			**Recurrent cyst**		
		**∑**	**Yes**	**No**	
		N (% column)	N (% row)	N (% row)	*p*-value
Thecaloscopy	Yes	48 (61.5)	5 (10.4)	43 (89.6)	0.0368
	No	30 (38.5)	9 (30.0)	21 (70.0)	
	**∑**	78	14 (17.9)	64 (82.1)	

**Table 2:** Obtained data from all 78 patients who underwent microsurgical resection of Tarlov cysts.

			∑	Thecaloscopy	No Thecaloscopy	*p*-Value	Recurrent Cyst	No Recurrent Cyst	*p*-Value
			N (%)	N (%)/Mean ± SD	N (%)/Mean ± SD		N (%)/Mean ± SD	N (%)/Mean ± SD	
Demographic information									
	Age [years]		78	48/50.0 ± 11.4	30/54.4 ± 9.0	0.077	14/53.1 ± 7.6	64/51.4 ± 11.3	0.590
	Gender		78						
		female	63 (80.8)	38 (60.3)	25 (39.7)	0.772	10 (15.9)	53 (84.1)	0.453
		male	15 (19.2)	10 (66.7)	5 (33.3)		4 (26.7)	11 (73.3)	
	Height [cm]		78	48/169.9 ± 7.3	30/179.6 ± 7.6	0.689	14/175.0 ± 7.2	64/169.2 ± 7.1	0.007
	Weight [kg]		78	48/66.8 ± 13.2	30/71.1 ± 13.8	0.168	14/73.4 ± 13.7	64/67.4 ± 13.4	0.136
	Body mass index		78	48/22.7 ± 3.7	30/23.9 ± 3.9	0.189	14/23.6 ± 3.5	64/23.1 ± 3.9	0.674
Clinical parameters									
	Symptoms duration [years]		77	47/6.8 ± 9.9	30/2.5 ± 3.3	0.025	14/3.5 ± 4.5	63/5.5 ± 8.8	0.422
	Pain level (VAS)								
		pre-OP	78	48/8.0 ± 2.0	30/8.0 ± 1.3	0.960	14/8.4 ± 0.9	64/7.9 ± 1.9	0.420
		Follow up	78	48/4.5 ± 2.7	30/5.2 ± 2.7	0.280	14/6.4 ± 2.2	64/4.4 ± 2.7	0.010
	Bladder or bowl dysfunction		78						
		Yes	46 (59.0)	29 (63.0)	17 (37.0)	0.815	5 (10.9)	41 (89.1)	0.072
		No	32 (41.0)	19 (59.4)	13 (40.6)		9 (28.1)	23 (71.8)	
	Dysesthesia		78						
		Yes	59 (75.6)	34 (57.6)	25 (42.4)	0.282	10 (16.9)	49 (83.1)	0.735
		No	19 (24.4)	14 (73.7)	5 (26.3)		4 (21.1)	15 (78.9)	
	Sexual dysfunction		78						
		Yes	8 (10.3)	5 (62.5)	3 (37.5)	1.000	2 (25.0)	6 (75.0)	0.629
		No	70 (89.7)	43 (55.1)	27 (44.9)		12 (17.1)	58 (82.9)	
	Symptoms after surgery		78						
		Improved	55 (70.5)	35 (63.6)	20 (36,4)	0.582	5 (9.1)	50 (90.9)	0.006
		Equal	18 (23.1)	11 (61.1)	7 (38.9)		7 (41.2)	10 (58.8)	
		Worsened	5 (6.4)	2 (40.0)	3 (60.0)		2 (33.3)	4 (66.7)	
	Residual symptoms								
		Yes	63 (80.8)	37 (58.7)	26 (41.3)	0.383	11 (17.5)	52 (82.5)	1.000
		No	15 (19.2)	11 (73.3)	4 (26.7)		3 (20.0)	12 (80.0)	
Cyst characteristics									
	Number		78	48/2.1 ± 1.2	30/2.6 ± 1.6	0.143	14/2.9 ± 1.5	64/2.2 ± 1.3	0.103
	Size [mm]								
		max	75	47/23.0 ± 15.2	28/26.3 ± 14.0	0.357	14/25.9 ± 8.0	61/23.0 ± 16.0	0.637
		mean	75	47/20.0 ± 15.8	28/22.3 ± 16.4	0.592	14/18.4 ± 6.0	61/21.3 ± 17.1	0.136
	Bilateral		78						
		Yes	34 (43.6)	26 (76.5)	8 (23.5)	0.020	13 (27.1)	35 (72.9)	0.002
		No	44 (56.4)	22 (50.0)	22 (50.0)		1 (3.3)	29 (96.7)	
	Multisegmental		77						
		Yes	43 (55.8)	24 (55.8)	19 (44.2)	0.238	10 (23.3)	33 (76.7)	0.129
		No	34 (44.2)	24 (70.6)	10 (29.4)		4 (11.8)	31 (88.2)	
	Bone erosion		78						
		Yes	54 (43.6)	37 (68.8)	17 (31.5)	0.078	8 (14.8)	46 (85.2)	0.342
		No	24 (56.4)	11 (45.8)	13 (54.2)		6 (25.0)	18 (75.0)	

## Data Availability

The datasets generated during and/or analyzed during the current study are available from the corresponding author on reasonable request.
